# Retina-like Computational Ghost Imaging for an Axially Moving Target

**DOI:** 10.3390/s22114290

**Published:** 2022-06-05

**Authors:** Yingqiang Zhang, Jie Cao, Huan Cui, Dong Zhou, Bin Han, Qun Hao

**Affiliations:** 1 Key Laboratory of Biomimetic Robots and Systems, School of Optics and Photonics, Beijing Institute of Technology, Ministry of Education, Beijing 100081, China; 3120200652@bit.edu.cn (Y.Z.); 3120190619@bit.edu.cn (H.C.); 3120195343@bit.edu.cn (D.Z.); 3120170330@bit.edu.cn (B.H.); 2 Yangtze Delta Region Academy, Beijing Institute of Technology, Jiaxing 314003, China

**Keywords:** computational ghost imaging, retina-like imaging, target axial motion, image reconstruction technique

## Abstract

Unlike traditional optical imaging schemes, computational ghost imaging (CGI) provides a way to reconstruct images with the spatial distribution information of illumination patterns and the light intensity collected by a single-pixel detector or bucket detector. Compared with stationary scenes, the relative motion between the target and the imaging system in a dynamic scene causes the degradation of reconstructed images. Therefore, we propose a time-variant retina-like computational ghost imaging method for axially moving targets. The illuminated patterns are specially designed with retina-like structures, and the radii of foveal region can be modified according to the axial movement of target. By using the time-variant retina-like patterns and compressive sensing algorithms, high-quality imaging results are obtained. Experimental verification has shown its effectiveness in improving the reconstruction quality of axially moving targets. The proposed method retains the inherent merits of CGI and provides a useful reference for high-quality GI reconstruction of a moving target.

## 1. Introduction

Ghost imaging (GI), also known as single-pixel imaging, has attracted broad attention in recent years [1,2,3,4]. The traditional ghost imaging system consists of two optical paths: the reference light path and the signal light path. In the reference light path, the beam propagates freely, and the intensity distribution information is detected by a detector with spatial resolution. In the signal light path, the signal beam illuminates the target, and the transmitted or reflected light is collected by a bucket detector without spatial resolution. The image is computed through correlation between the measurements of two light paths, rather than each one separately [5,6]. In 2008, computational ghost imaging (CGI) [7,8] was proposed, which omitted the reference path via a spatial light modulator or digital micromirror device (DMD) that is actively used to control and compute predictable illumination patterns. The single-arm structure improves the practicality of GI in fluorescence imaging [9], terahertz imaging [10,11], multispectral imaging [12,13,14,15,16,17], three-dimensional imaging [18,19,20,21,22], and scattering medium imaging [23,24,25].

Undoubtedly, for the application of GI, the development trend is bound to be from stationary targets to moving targets. Compared with stationary targets, moving targets pose a greater challenge to GI technology. When the target moves relatively toward the imaging system, the reconstructed image suffers resolution degradation, as well as image quality decline. Eliminating the image degradation caused by target motion is worthy of further research.

Generally, the relative motion between the target and the imaging system can be divided into two cases: tangential motion perpendicular to the optical axis and axial motion along the optical axis. In 2011, Li et al. [26] took the idea of quasistatic estimation in target motion. Within a small segmental interval, the target can be regarded as stationary. Zhang et al. [27] proposed a Fourier transform ghost diffraction imaging method to eliminate the image degradation caused by a shaking target. Li et al. [28] successfully reconstructed the tangentially moving target by translationally compensating for the light-intensity distribution on the reference light path. In 2020, Yang et al. [29] proposed a tracking compensation method to improve the degradation of target motion. By shifting or rotating the illumination patterns preloaded on the DMD according to the precise estimate of the moving target’s motion track, they achieved high-quality reconstruction. Gong et al. [30] investigated the influence of the axial correlation depth-of-light field in GI applications. In 2015, Li et al. [31], based on resizing speckle patterns and velocity retrieval, experimentally reconstructed an axially moving target in a dual-arm GI system. In 2017, the high-order GI theory was applied to investigate the impact of light field high-order intensity correlation on axially moving target reconstruction [32]. Compared with the tangential motion, there are relatively fewer discussions of the target’s axial motion in current studies, many of which are still based on a dual-arm ghost imaging system.

Inspired by the structure of the human eye, retina-like patterns are designed to balance high-resolution and high-imaging efficiency [33,34]. In our previous work [35], retina-like structure patterns were utilized in three-dimensional CGI, which has been proven effective in compressing redundant information and speeding up the imaging process while improving the reconstruction quality of the foveal region of interest. In this paper, we propose a time-variant retina-like computational ghost imaging (VRGI) method that uses retina-like patterns with changeable foveal region radii to reconstruct an axially moving target while controlling the target well in the light field during the movement process. There is no need to predict the precise motion track of targets or adjust the DMD in advance. Meanwhile, compared with the dual-arm GI system, our method is easier to implement and can restore the imaging quality of moving targets without redundant hardware requirements.

## 2. Methods

In a CGI system, a series of illumination patterns are projected onto the target object. The reflected or transmitted light intensity of the target is collected by the single-pixel detector. The final image is reconstructed by correlating the information of illumination patterns and their corresponding light-intensity measurements.

The measurement process is described as follows:

(1) $$S_{m}=\sum_{x,y}U_{m}\left(x,y\right)O\left(x,y\right)$$

where *U_m_*(*x*,*y*) represents a sequence of illumination patterns, *m* is the pattern sequence number, and (*x*,*y*) represents the 2D Cartesian coordinates. *O*(*x*,*y*) is the target. Thus, the corresponding collected light intensity is denoted as *S_m_*.

When considering the reconstruction algorithm, it has been proven that the compressed sensing (CS) algorithm [36], also known as compressed sampling, has better performance than the conventional second-order correlation algorithm. It provides an alternative approach to data acquisition and compression that reduces the number of required measurements by the aid of the redundant structure of images, and, therefore, has drawn much research attention.

Specifically, the data collection in CS can be described as:

(2) y=Ab

where *y* is the measurement vector, *A* is the vectorized representation of the projected patterns, and *b* is a vector, which denotes the target scene that is assumed to be sparse. The reconstruction process is to calculate *b* from the pattern matrix *A* and the corresponding measurement *y*.

At present, CS algorithms mainly include *l*_1_ minimization [37], greedy algorithms [38], and TV minimization [39]. The total variation (TV) regularization algorithm, which transforms the image reconstruction problem into a constrained optimization problem, is selected in this paper as our reconstruction algorithm, as it performs better in preserving the boundaries or edges of the reconstructed images [40]. By exploiting TV solver, a sparse image may still be reconstructed with a relatively high quality even though the number of measurements is deemed insufficient.

Mathematically, *c* represents the gradient of an image, and *G* is the gradient calculation matrix. $U^{\prime }\in R^{M*a}$ is the illumination pattern (with *M* being the projection number of the illumination patterns and each pattern comprising *a* = x × *y* pixels); $O^{\prime }\in R^{a*1}$ represents the target scene that aligned as a vector; and $S^{\prime }\in R^{M*1}$ is the collected light intensity. The *l*_1_ norm is used to calculate the total variation of the image, so the optimization model becomes:

(3) $$\begin{array}{ll} \min & {\left\|c\right\|}_{l_{1}}\\ s.t. & GO^{\prime }=c\\ & U^{\prime }O^{\prime }=S^{\prime } \end{array}$$

The retina has high resolution in the foveal region and low resolution in the edge region. This characteristic can effectively enhance the quality of the reconstructed images in the foveal region of interest. On this basis, the CGI method with retina-like patterns (or the so-called RGI method) uses retina-like patterns with space-variant resolution instead of random patterns with uniform resolution.

With reference to our previous studies on retina-like structures, the geometry of the retina-like patterns can be expressed as follows:

(4) $$\left\{ \begin{array}{l} r_{p}+1=r\times \varepsilon^{p}\\ \varepsilon =\frac{1+\sin(\pi /Q)}{1-\sin(\pi /Q)}\\ r_{1}=\frac{r_{0}}{1-\sin(\pi /Q)}\\ \theta_{q}=q\times \frac{2\pi }{Q}(q=1,2,3\ldots Q)\\ \zeta_{p}=\log_{\varepsilon }(rp)=\log_{\varepsilon }(r1)+p-1(p=1,2,3\ldots P) \end{array}\right.$$

Each pattern includes *P* rings, and each ring consists of *Q* pixels. *r_p_* is the radius of the *p_th_* ring; *θ_q_* is the degree of the *q* sector; and *ε* is the increasing coefficient. In the foveal area, retina-like patterns are nested with high-resolution speckles, which are the same as random patterns, whereas in the peripheral area, they have low-resolution speckles, and the resolution decreases with the increase in eccentricity.

Compared with that of a stationary target, the image degradation caused by a target in motion is a nonnegligible problem in CGI research on moving targets. In the case of axially moving targets, the RGI method can control the target well in the spatially fixed light field during the process of movement instead of adjusting the whole light field as the target is moving. The target can always be illuminated by the foveal region of the retina-like patterns.

However, the projection size of the foveal region of the retina-like patterns changes at different positions along the axis. The farther away from the optical system it is, the larger the projection area. To maintain the stability of the moving target in the light field of illuminated patterns, the proportion of the projection area of the foveal region occupied by the target should be kept unchanged as much as possible as the target moves back and forth along the axis. The foveal region of the illuminated retina-like patterns should be larger when the target is moving closer to the imaging system.

Focusing on the reconstruction of axially moving targets, the VRGI method adjusts the radius of the foveal region of the retina-like patterns, according to the specific motion parameters of the target and the information of the scene, to maintain the proportion of the target in the foveal region during the movement.

Based on a retina-like pattern with space-variant resolution, as shown in Equation (4), we change the radius *r*_0_ of the foveal region according to the axially moving velocity *v*, the moving distance (expressed as *v* × *t*) of the target, the size of the target (expressed as *d*), and *D,* which is the distance between the target and the optical projection system. The mathematical formula can be expressed as follows:

(5) $$r_{0}(i)=r_{0}(1)+v\times t(i)\times (d/D)$$

where *r*_0_(*i*) is the radius of the foveal region of the *i*-th retina-like pattern, *v* is the axial velocity of the target, *t*(*i*) is the moving time corresponding to the *i*-th speckle, *d* is the size of the target, and *D* is the distance between the target and the projection system. We set different increments according to different sampling measurements so that the radius of the foveal region of the retina-like pattern can be adjusted evenly during the process of movement.

## 3. Experimental Results

**Figure 1:**
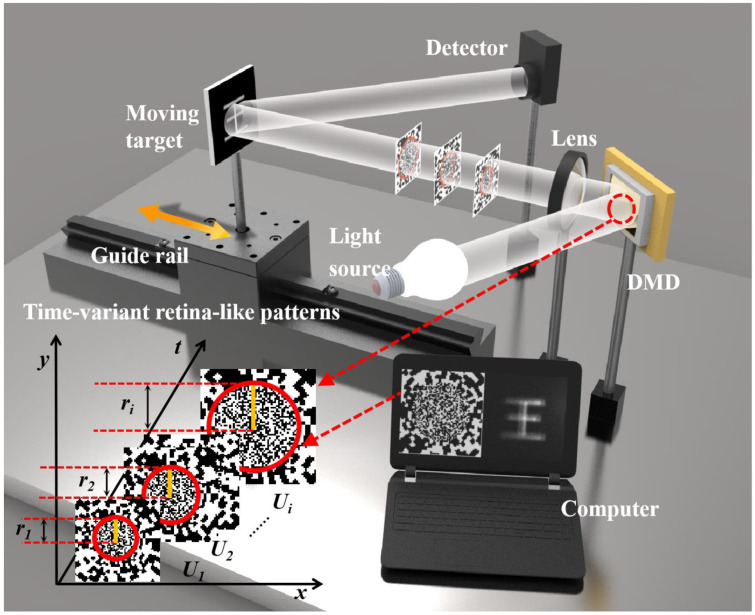
Experimental setup.

To implement the proposed method, we built the experimental setup, as shown in Figure 1. An LED light source operating at 400~750 nm (@20 W) uniformly illuminates the DMD (Texas Instruments DLP Discovery 4100 development kit, Texas Instruments, US). The maximum binary pattern refreshing rate of the DMD is up to 22 kHz, the size of the DMD is 1024 × 768 pixels and the micromirror pitch is 13.68 μm. The DMD projects preloaded patterns to the target through a projection lens. The target in the experiment is a symmetrical Chinese character printed on paper. It is placed on a one-dimensional guide rail and moves back and forth along the optical axis at a constant speed, *v*. The distance and speed of the movement is controlled by a stepper motor. A photodetector (Thorlabs PDA36A, Newton, NJ, USA) with an active area of 13 mm^2^ functions as a bucket detector and measures the reflected light intensities of the target. A data acquisition board (PICO6404E, Pico Technology, Rowley Park, UK) acquires and transfers the measured intensities to a computer for reconstruction.

In our experiment, the resolution of the retina-like patterns is 64 × 64, and the ratio *d*/*D* of the target size *d* to the distance *D* between the target and the optical projection system is set at 0.0625. The target moves at a uniform velocity toward the optical system along the optical axis. The total moving distance is 5 mm. To compare the reconstruction results of the axially moving target at different *v* values, we set the moving velocities as 0.5 mm/s, 1 mm/s, 1.5 mm/s, and 2 mm/s.

According to Equation (5), retina-like patterns with uniformly varying foveal region radii are generated according to the number of measurements that are needed. The radius of the foveal region varies [21,23]. We reasonably set the projection time of illumination patterns to make it equal to the motion time of the target and ensure that the target would not move beyond the illumination field.

**Figure 2:**
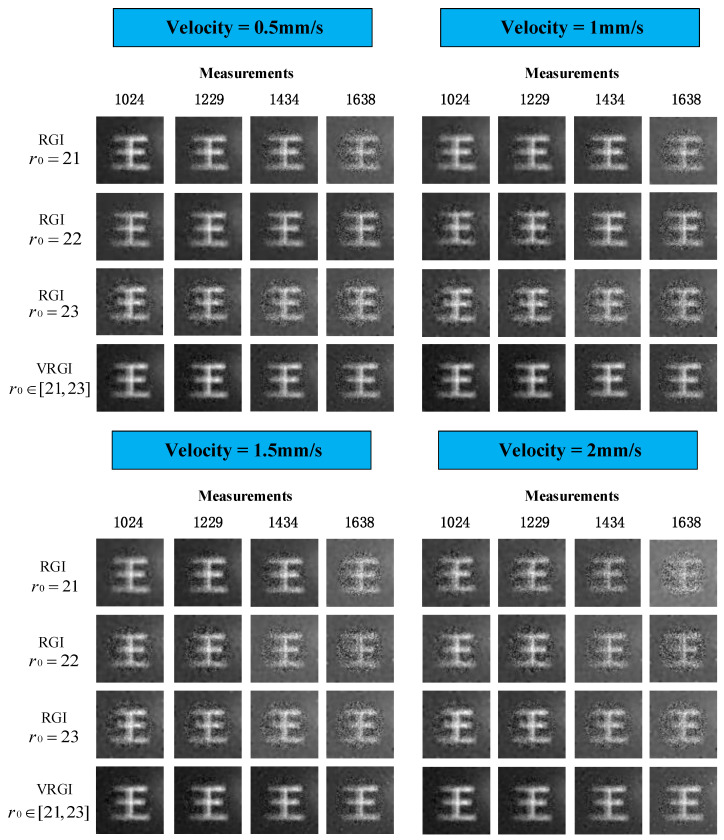
Reconstruction of an axially moving target by RGI and VRGI with different measurements and differen t velocities.

Since the compressed sensing algorithm enables the GI reconstruction of an *N* pixel image with much fewer measurements than *N*, we set the sampling numbers as 1024, 1229, 1434, and 1638 here. The experiments are performed with these four different sampling measurements and different moving velocities, and the reconstruction results retrieved by TV regularization algorithm are shown in Figure 2.

The experimental results compare the VRGI with variable foveal region radii and the traditional RGI method with an unchanged foveal region radius (a minimum value of 21, a middle value of 22, and a maximum value of 23). For the 1024, 1229, 1434, and 1638 measurements, all the reconstructed images become blurred with increasing moving velocity, but generally, VRGI outperforms traditional RGI no matter how fast the target moves along the optical axis, with a more distinguishable and detailed structure of the character.

For quantitative comparison, the peak signal-to-noise ratio (PSNR) [41] was used as the criterion. The PSNR is defined as follows:

(6) $$\left\{ \begin{array}{l} \mathrm{PSNR}=10\log_{10}\frac{{\left(2^{k}-1\right)}^{2}}{\mathrm{MSE}}\\ MSE=\frac{1}{a}\sum {}_{x,y}{\left(O^{\prime }\left(x,y\right)-O\left(x,y\right)\right)}^{2} \end{array}\right.$$

where *k* represents the number of bits, and *O*(*x*,*y*) and *O′*(*x*,*y*) represent the original image and reconstructed image, respectively. MSE stands for the mean square error.

**Figure 3:**
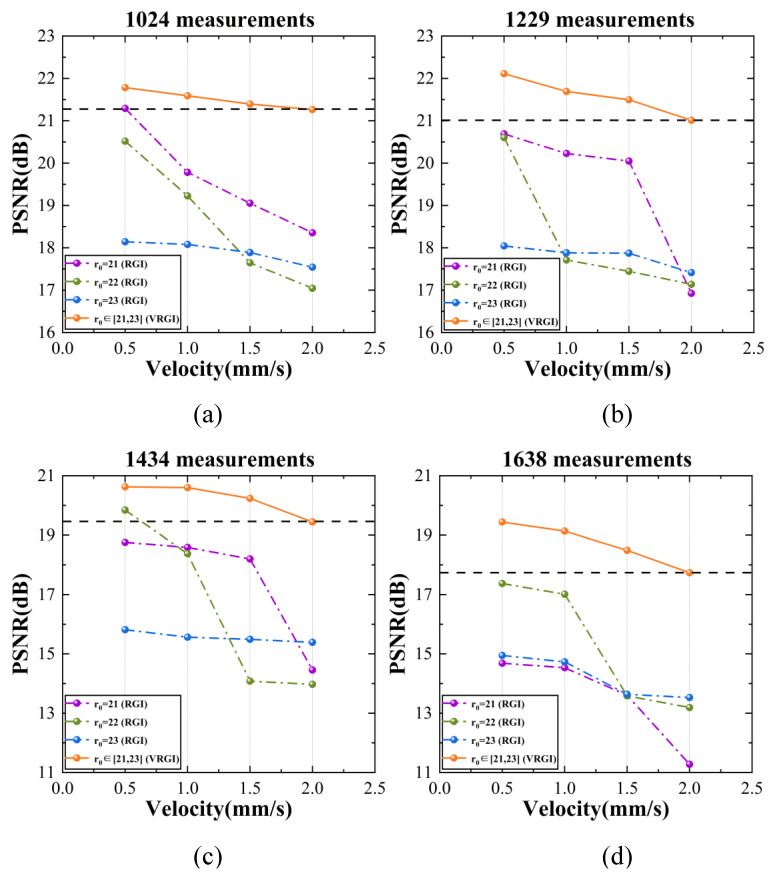
PSNR values of RGI and VRGI images: (**a**) PSNR of RGI and VRGI images at different velocities with 1024 measurements; (**b**) PSNR of RGI and VRGI images at different velocities with 1229 measurements; (**c**) PSNR of RGI and VRGI images at different velocities with 1434 measurements; and (**d**) PSNR of RGI and VRGI images at different velocities with 1638 measurements.

Figure 3 shows the quantitative comparison of PSNR values for 1024, 1229, 1434, and 1638 measurements. Generally, there is a linear relationship between the moving velocity and reconstruction quality, the faster the target moves, the worse the image degrades, with inevitable blurriness and a higher noise level. However, changing the foveal region radius *r*_0_ according to the movement could reduce the negative influence of image degradation caused by target motion. The PSNR values of the proposed method are obviously higher than those of the other three groups of traditional RGI method.

As shown in Figure 3a, for 1024 measurements, the moving velocity of the target increases from 0.5 mm/s to 2 mm/s, and the PSNR values of VRGI results only decrease from 21.78 dB to 21.26 dB. For the traditional RGI method with an unchanged radius of the foveal region, the best reconstruction quality can reach only 21.29 dB at the slowest velocity and can reach only 18.35 dB when the moving velocity increased to 2 mm/s. The reconstruction quality of VRGI at the fastest velocity is equivalent to or even better than that of RGI at the slowest velocity. In our experiment, when the velocity of the moving target increases to 2 mm/s, the reconstruction performance of RGI with *r*_0_ = 21 is unsatisfactory for 1638 measurements such that the target can hardly be identified, as shown in Figure 2. Obviously, 2 mm/s has not reached the velocity limit of VRGI method, even though it also suffers image quality decline as the target moves faster.

Moreover, patterns with different *r*_0_ values show different adaptability to the increase in the target’s moving velocity. RGI with *r*_0_ = 21 is greatly affected by it, while RGI with *r*_0_ = 23 is relatively stable. However, this does not mean that the size of the foveal region and reconstruction quality can be simply illustrated by an absolute linear relationship, and the influence of different measurements should also be considered. Under low measurement conditions, the PSNR values of RGI images with *r*_0_ = 23 change steadily as the velocity increases, but the reconstructed results do not perform better than those of small *r*_0_ patterns.

In addition, the TV solver is applied here to decrease the sampling ratio, and we indeed obtain high-quality images under low measurement conditions, as shown in Figure 3a,b. However, when the measurements gradually reach 1638, the redundant calculation makes the noise level even higher and cannot achieve an effective utilization.

Therefore, a reasonable selection of the variation range of *r*_0_ and sampling measurements are needed, according to the scene information and target motion parameters in practical applications, to achieve the optimal match.

## 4. Conclusions

The image degradation caused by relative motion between the target and the imaging system decreases the imaging resolution and affects the reconstruction quality in ghost imaging. In this paper, we proposed and demonstrated a time-variant retina-like computational ghost imaging strategy for axially moving targets to suppress the image degradation caused by target motion in GI applications. In comparison with axially moving schemes conducted in a dual-arm system in previous studies, the proposed method can maintain the inherent advantages of computational ghost imaging and reconstruct the target in a single-arm system without redundant hardware requirements. The illumination patterns projected by DMD are designed with retina-like structures, and the radii of foveal region can be modified according to the moving process of the target. The experimental results demonstrate that the proposed method has better performance in reconstructing axially moving targets than the traditional RGI method with retina-like patterns containing an unchanged foveal region size. By reasonably considering the size of the foveal region in retina-like patterns and the sampling range of measurements, the most effective match is achievable. Although the proposed method can restrain the influence of image degradation caused by motion to a certain extent, there is still a possibility for optimization. Further improvement can be achieved by adapting a more efficient reconstruction algorithm, as well as filling our retina-like structure with other forms of illumination patterns that contain more information or sparsity prior of targets. It is believed that the proposed method will broaden the applications in real-time imaging, remote sensing, unmanned driving, and other fields that need to estimate scene information based on images.

## Data Availability

Data underlying the results presented in this paper are not publicly available at this time but may be obtained from the authors upon reasonable request.
